# Multiplexed LC-MS analysis reveals novel insights into grapevine defense mechanisms by expanding metabolome coverage

**DOI:** 10.1007/s11306-026-02410-y

**Published:** 2026-03-07

**Authors:** Pedro G. Vásquez-Ocmín, Amelie Pérez, Ana Romeo-Oliván, Virginie Puech-Pages, Sylvie Fournier, Bernard Dumas, Alban Jacques, Guillaume Marti

**Affiliations:** 1 Laboratoire de Recherche en Sciences Végétales, Université de Toulouse, CNRS, Toulouse INP, Toulouse, France; 2 Metatoul-AgromiX Platform, LRSV, Université de Toulouse, CNRS, Toulouse INP, Toulouse, France; 3 MetaboHUB-MetaToul, National Infrastructure of Metabolomics and Fluxomics, Toulouse, France; 4 Université de Toulouse, Ecole d’Ingénieurs de PURPAN, PPGV, Toulouse, France

**Keywords:** Metabolomics, Lipidomics, Oxylipins, Esca

## Abstract

**Introduction:**

Grapevine trunk diseases (GTDs), such as esca, pose a major threat to viticulture worldwide and are associated with complex biochemical responses in woody tissues. Comprehensive metabolome coverage remains a challenge, as conventional methods often overlook non-polar metabolites critical to plant defense mechanisms.

**Objectives:**

This study aimed to expand metabolome and lipidome coverage of grapevine wood by integrating complementary LC-MS approaches, in order to identify metabolic signatures linked to pathogenic fungi and to a biocontrol agent.

**Methods:**

Woody tissues of *Vitis vinifera* cv. Cabernet-Sauvignon were inoculated with *Phaeomoniella chlamydospora*, *Phaeoacremonium minimum*, and/or the biocontrol fungus *Trichoderma atroviride* (Vintec^®^). A biphasic extraction was coupled with three orthogonal LC-MS methods—reverse-phase (RP), hydrophilic interaction chromatography (HILIC), and lipidomics-focused RP. Data were processed through the MSCleanR workflow and integrated using the DIABLO multi-block statistical framework. Compound classification was performed with NPClassifier.

**Results:**

The multiplexed strategy enabled the annotation of 1,425 unique features, representing an 83% increase compared to previous studies. Distinct metabolomic and lipidomic signatures were associated with fungal infection and biocontrol treatments. Lipidomic analysis highlighted oxidized fatty acids (oxylipins) —specifically hydroxy-eicosatetraenoic acids (13-HETE, 16(R)-HETE, and 11(R)-HETE)—as potential signaling molecules in defense responses. NPClassifier revealed diverse biosynthetic classes, including phenylpropanoids, terpenoids, and sphingolipids, underscoring the chemical heterogeneity of grapevine responses.

**Conclusion:**

This multiplexed LC-MS workflow provides a versatile analytical pipeline for untargeted metabolomics and lipidomics in plants. By integrating complementary methods, the study uncovered novel biomarkers of grapevine defense, particularly oxylipins, emphasizing the critical role of lipidomics in deciphering plant–pathogen interactions.

**Supplementary Information:**

The online version contains supplementary material available at 10.1007/s11306-026-02410-y.

## Introduction

Viticulture is one of the most important agricultural practices globally. The cultivation of *Vitis vinifera* L. holds significant economic value; however, it is also the most well-known and disease-prone vine species. Grapevine trunk diseases (GTD), such as esca, BDA or Eutypa dieback, represent a great concern in viticulture worldwide. Esca is a vascular wilting disease that attacks the perennial organs of the vine, causing substantial necrosis in the trunk and cordon through the slow and systemic development of pathogenic fungi such as *Phaeomoniella chlamydospora*, *Phaeoacremonium minimum*, *Fomitiporia mediterranea* and Botryosphaeriaceae species (Larignon & Dubos, 1997; Mostert, 2006; Ouadi et al., 2019). It is the first cause of vine decline. The disease induces a decline in plants, potentially leading to their death. The physiological mechanisms that lead to foliar symptoms are not well understood. To better understand these mechanisms and address this major crisis, further study is necessary. The lack of effective treatment and control has caused significant concern in the wine industry. Previously, sodium arsenite was the only pesticide registered in Europe to control esca, but it was banned in the early 2000 s due to its toxicity to growers and the environment (Philippe Larignon et al., 2014). Currently, there is no completely effective curative method against esca. However, the search for new sources of biocides and their potential active compounds is the objective of several research groups worldwide.

Metabolomics and lipidomics play a crucial role in investigating organismal metabolomes to discover novel biocontrol agents and their biomarkers. Metabolomics offers a comprehensive profile of metabolites (< 1.5 KDa) within an organism, providing insights into biochemical pathways and physiological responses under various conditions (Shen et al., 2023; Tenenboim & Brotman, 2016; Tinte et al., 2021), while lipidomics focuses on lipid-specific roles in processes such as cellular signaling, energy metabolism, and defense (Kehelpannala et al., 2021; Liu et al., 2024). These approaches, particularly through advanced LC-MS techniques, enable the detection and quantification of diverse metabolites, facilitating the identification of biomarkers that signal the presence and efficacy of biocontrol agents (Mesguida et al., 2025).

Recent work showed metabolomic reprograming in grapevine infected leaves (Lemaitre-Guillier et al., 2020; Goufo & Cortez, 2021; Weiller et al., 2024) and wood (Galarneau et al., 2021; Labois et al., 2020) in response to GTD-associated pathogens. The initial extraction method predominantly yields metabolites of medium to high polarity, such as phenols and stilbenes, which are identified as the main biomarkers (Chervin et al., 2022). However, few publications have addressed the limitations of this extraction approach, particularly its omission of non-polar metabolites in biomarker discovery.

Our group has expertise in metabolomic approaches to understanding plant responses to biotic and abiotic stresses. Additionally, we develop comprehensive workflows using LC-MS and statistical analyses, allowing for the detection and annotation of major biomarkers (Vásquez-Ocmín, Cojean, et al. 2023; Vásquez-Ocmín et al. 2021). We previously studied the responses of grapevine wood attacked by both pathogens *P. chlamydospora* (P.ch), *P. minimum* (P. min), with and without treatments using the commercial biocontrol Vintec^®^. We identified the optimal kinetic inoculation period (3 weeks). We identified metabolic biomarkers to monitor the efficacy of the Vintec^®^ treatment and analyze its impact on the physiology of the plant attacked by P.ch and P.min (Chervin et al. 2022). Dereplication highlighted the role of glycerophospholipids and polyphenols (mainly stilbenoids) in all plant-microbes interactions. By the use of Vintec pretreatment we revealed enhanced accumulation of pterostillbene, resveratrol and oxyresveratrol. The antifungal activity of these biomarkers, evaluated in vitro on *Phaeomonia chlamydospora* and *Botrytis cinerea*, suggests that these compounds may play a role in limiting the development of esca pathogens in the plant (Chervin et al. 2022).

In the present study, we chose to extract highly polar to non-polar lipids using a biphasic solvent extraction and to develop orthogonal LC-MS approaches to characterize a large part of the grapevine metabolome. The liquid and stationary phases of the chromatography were adapted to metabolomic (RP and HILIC) and lipidomic analyses (RP). In this context, the integration of metabolomics and lipidomics is crucial for the discovery of new biocontrol agents and their associated biomarkers. This work highlights, through these multiplexed approaches, the production or inhibition of certain biomarkers by grapevine wood in the presence of pathogenic fungi and the biocontrol fungus Vintec^®^. To our knowledge, the implementation of this type of multiplexed analyses has not been used so far.

## Materials and methods

### Plant culture, inoculation and extraction

Plant material consisted in two-node cuttings of *V. vinifera* cv. Cabernet-Sauvignon clone 15 from Daydé Nurseries (Occitanie, France). The disinfection procedure, plant culture conditions, fungal inoculation procedure and sample collection (3 weeks post-inoculation) were the same as those described previously by Chervin et al., 2022 (Chervin et al., 2022). The following conditions were studied: (1) Injured/Not inoculated (INi); (2) Biocontrol *Trichoderma* Vintec^®^ (Injured/Vintec^®^, IV); (3) *P. minimum* (Pmin); *P. chlamydospora* (Pch) (Injured/*P. chlamydospora* + *P. minimum*, IPP); (4) *P. minimum*, *P. chlamydospora*, Vintec^®^ (Injured/P. *chlamydospora* + *P. minimum* + Vintec^®^, IVPP).

Freeze-dried wood samples (~ 1 cm from inoculation area) were ground under liquid nitrogen using a Retch POWTEQ GT 300 ball mill. Approximately 100 mg of the resulting powder was first extracted with 700 µL of methyl-*tert* butyl ether (MtBE)/methanol (MeOH) (75:25, v/v) mixture, then vortex and sonicated for 3 min. Afterward, 700 µL a water/MeOH (75:25, v/v) mixture was added, followed by vortexing and sonication for another 3 min. The extract was centrifuged for 3 min at 5 °C at approximately 14,000 RPM. The resulting upper (apolar) and lower (polar) phases were collected into pre-weighed tubes and dried overnight in a SpeedVac at 37 °C (Vásquez-Ocmín, Marti, et al. 2023). The dried polar phase residue was reconstituted in 80:20 MeOH/water, and the apolar phase residue was reconstituted in 100% MeOH to a final concentration of 5 mg/mL for LC-MS analysis. A quality control (QC) sample was prepared by pooling equal aliquots from each extract.

### Metabolome analyses

#### Untargeted metabolomics

A Q Exactive™ Plus Hybrid Quadrupole-Orbitrap™ mass spectrometer equipped with a Heated Electrospray Ionization (HESI-II) probe, coupled to an U-HPLC Vanquish H (Thermo Fisher Scientific, Hemel Hempstead, U.K.) was utilized. Mass detection was performed in positive ionization (PI) and negative ionization (NI) modes with a resolution of 30,000 [Fullwidth at half-maximum (fwhm) at 400 *m/z*] for MS1 and 17,500 with an automatic gain control (AGC) target of 1 × 10^6^ for MS1 and 1 × 10^5^ for MS2. The ionization spray voltage was set to 3.5 kV for PI and 2.5 kV for NI, and the capillary temperature was set to 256 °C for both modes. The mass window ranged from 100 to 1500 *m/z*. Each full MS scan was followed by acquisition of data-dependent MS/MS spectra for the six most intense ions (top 6) using progressive normalized collision energies of 20, 40, and 60 eV.

Reverse-phase (RP) chromatographic separation was performed on a Luna Omega Polar C18 UHPLC column (150 mm 2.1 mm, 1.6 μm) with a pre-column (Phenomenex, Sartrouville, France). For the mobile phase gradient used water containing 0.05% formic acid (A) and acetonitrile containing 0.05% formic acid (B). A constant flow gradient method of 0.4 mL/min was applied under the following conditions: 2% B for 0.5 min, increasing to 70% B over 10 min, held at 98% B for 2.1 min, then equilibrated with 98% A for 1.4 min. The column oven temperature was set at 40 °C, the autosampler temperature was set at 5 °C, and the injection volume was set to 1 µL.

Hydrophilic interaction liquid chromatography (HILIC) separation was performed on a ZIC^®^-HILIC UHPLC column (150 mm 2.1 mm, 3.5 μm) with a pre-column (Merck). The mobile phase gradient used water containing 10 mM ammonium formate (A) and acetonitrile containing 10 mM ammonium formate (B). A constant flow gradient method of 0.3 mL/min was applied under the following conditions: 100% B for 0.5 min, decreasing to 50% B over 8.5 min, held at 50% B for 2 min, then equilibrated with 100% B for 8.9 min. The column oven temperature was set to 40 °C, the autosampler temperature was set to 5 °C, and the injection volume was set to 1 µL.

#### Untargeted lipidomics

Lipidomics analysis was performed on the same LC-MS system. Chromatographic separation was achieved using an Acquity UPLC CSH C18 column (100 mm, 2.1 mm, 1.7 μm) with a pre-column (Waters, Massachusetts, USA). The mobile phase gradient used acetonitrile (ACN)/water (60:40, v/v) with 10 mM ammonium formate and 0.1% formic acid (A) and isopropanol/ACN (90:10, v/v) with 10 mM ammonium formate and 0.1% formic acid (B). A constant flow gradient method of 0.3 mL/min was applied under the following conditions: 40% B as the initial condition (curve 6), rising to 43% B in 2 min, then to 50% B in 0.1 min (curve 1), then rising to 54% B in 11.9 min, then to 70% B in 0.1 min (curve 1), then to 99% in 5.9 min, held at 99% B for 2 min, then equilibrated with 40% B for 2 min (curve 1). The column oven temperature was set to 55 °C, the autosampler temperature was set to 5 °C, and the injection volume was set to 1 µL.

#### Data mining process

The LC-MS data were processed according to the MSCleanR workflow (Fraisier-Vannier et al., 2020). Briefly, one positive ionization (PI) and one negative ionization (NI) batch were processed with MS-DIAL version 4.92 (Tsugawa et al., 2015). MS1 and MS2 tolerances were set to 0.01 and 0.05 Da, respectively, in centroid mode for each data set. Peaks were aligned to a reference (QC) with an RT tolerance of 0.2 min, a mass tolerance of 0.015 Da, and a minimum peak height detection at 5 × 10^5^ for all three methods. MS-DIAL data were deconvoluted with MS-CleanR by selecting all filters with a minimum void ratio set to 0.8 and a maximum relative standard deviation (RSD) set to 40%. The maximum mass difference for detection of functional relationships was set to 0.005 Da, and the maximum RT difference was set to 0.025 min. Pearson correlation links were used with a correlation of 0.8 and a p-value significance level of 0.05. Two peaks were retained in each cluster for further database requests, and the retained features were annotated with MS-FINDER version 3.52 (Tsugawa et al., 2016). MS1 and MS2 tolerances were set at 15 and 20 ppm, respectively. The formula detector was exclusively based on C, H, O, N, and P.

Four levels of compound annotation of multiple data sets were performed. Level 1 metabolite annotation was done using MS-DIAL with LC-MS/MS experimental data of internal AgromiX database of 500 compounds (retention time, exact mass and fragmentation). Level 2 metabolite annotation was performed based on FragHub (Dablanc et al., 2024) v 1.0 (https://zenodo.org/records/10837523) mass spectral records in.msp format. The databases were used for spectral matching by applying a dot product score of 800 (~ 50,000 compounds). Level 3 metabolite annotation was prioritized based on: i) a search using MSFinder for a match with compounds identified in the literature for grapevine, genus *Vitis* (genus level) and family Vitaceae (family level); genus *Trichoderma* (genus of the Vintec^®^ biocontrol); compiled from the Dictionary of Natural Products (version 28. 2, CRC press) and based on exact mass and in silico fragmentation. Remaining features were annotated using MS-FINDER integrated databases (HMDB, PlantCyc, NANPDB, COCONUT Lipid MAPS, FooDB, Natural Products Atlas and KNApSAcK) (generic level). Features with only partial match (dot product score < 800) were considered as spectral analog (level 4). A workflow using Knime 4.92 (Berthold et al., 2008) was applied for the final cleaning of compounds (duplicates) and annotation prioritization.

#### Statistical analysis

For data integration, we used the mixOmics R package (http://mixomics.org/) (Le Cao et al. 2016; Rohart et al., 2017). Three distinct data blocks, each comprising unique features (defined as pairs of *m/z* x RT signals), were integrated: metabolomics data obtained using RP chromatography, metabolomics data using HILIC chromatography and lipidomics data. Each LC-MS data set (*m/z* x RT x peak area) was normalized to TIC (total ion chromatogram) and scaled by unit variance. A preliminary unsupervised principal component analysis (PCA) was performed for each LC-MS method and for the combined data set. To highlight the overall correlation between the chemical fingerprints of the extracts by LC-MS, an arrow plot was generated using sPLS-DA (Sparse Partial Least Square Discriminant Analysis) from multiple blocks. Data integration, classification, feature selection and visualization were performed using DIABLO (Data Integration Analysis for Biomarker discovery using Latent cOmponents), a latent variable approaches for multi-omics studies. Venn diagram and sunburst were constructed using an internal python script and NPclassifier chemical ontology (Kim et al., 2021).

## Results and discussions

### Merging metabolomics and lipidomics to decipher the whole metabolome

**Fig. 1:**
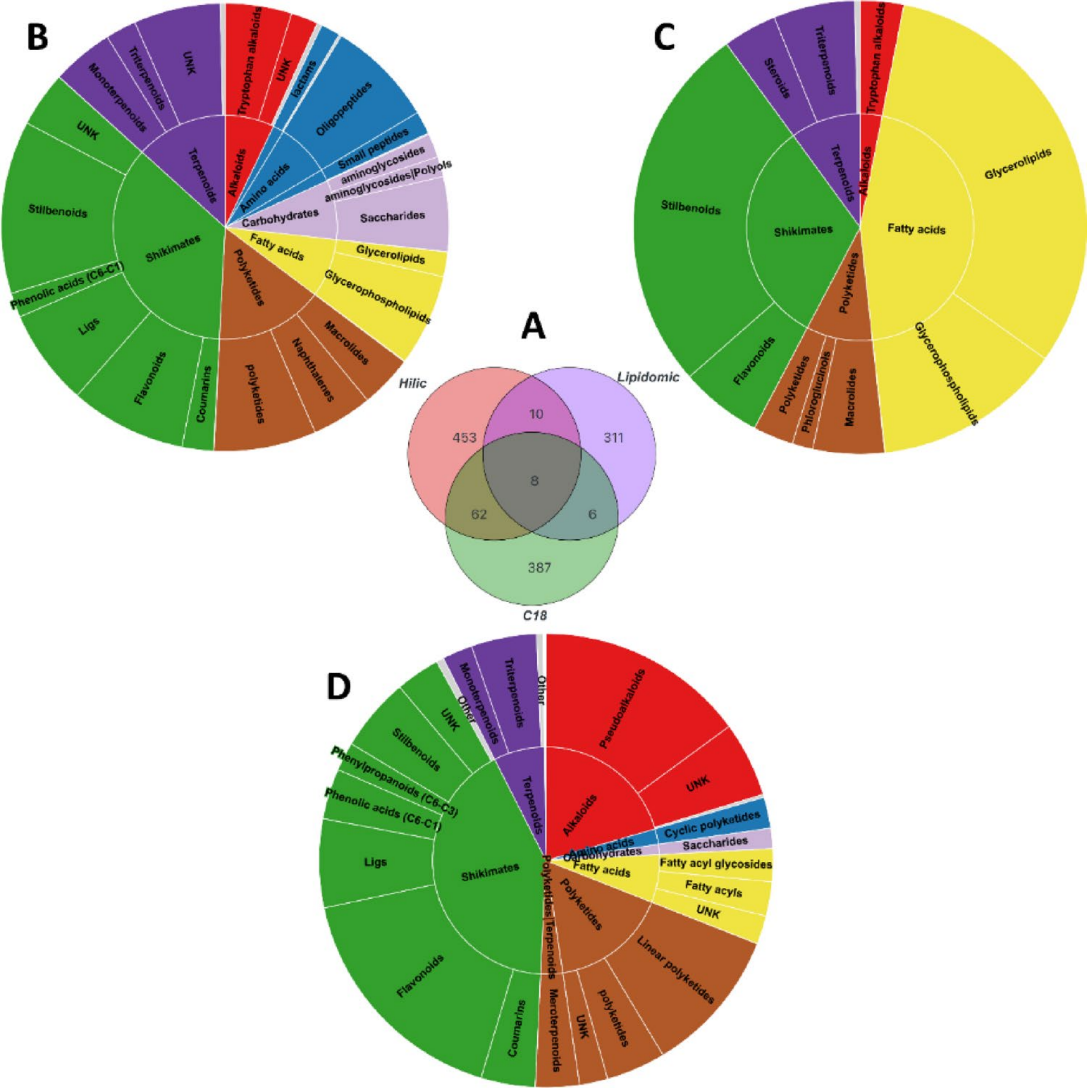
Enhanced metabolome coverage via three orthogonal LC-MS approaches. **A** Venn diagram representing the 1,425 features annotated using the three LC-MS analysis methods from the biphasic phase separation (metabolomics and lipidomics). **B** Sunburst diagram illustrating the major chemical classes identified through each type of LC-MS analysis, metabolomics-HILIC (M—HILIC), **C** lipidomics-RP (L-RP) and **D** metabolomics-RP (M-RP)

Wood of *V. vinifera* cv. Cabernet-Sauvignon were extracted using biphasic solvent separation, from several culture conditions: Injured/Not inoculated (INi); Biocontrol *Trichoderma* Vintec^®^ (Injured/Vintec^®^, IV); *P. minimum* (Pmin); *P. chlamydospora* (Pch) (Injured/*P. chlamydospora* + *P. minimum*, IPP); *P. minimum*, *P. chlamydospora*, Vintec^®^ (Injured/P. *chlamydospora* + *P. minimum* + Vintec^®^, IVPP), 3 weeks post-inoculation. The liquid and stationary phases of the chromatography were adapted to metabolomic phase analysis (RP and HILIC) and lipidomic phase analysis (RP), coupled to HR-MS. Features were extracted and processed. Our multiplexed analytical approach enabled the identification of 1,425 unique features (*m/z* × RT), representing an 83.2% increase in detected metabolites compared to the study by Chervin et al. (2022), which reported 239 features (Chervin et al., 2022). Specially, we identified 459 features for RP metabolomics, 577 features for HILIC metabolomics, and 353 features for RP lipidomics. As shown in Fig. 1A and 387 unique metabolites were identified exclusively in the RP metabolomics analysis (84.3% specificity), 453 for HILIC metabolomics (78.5% specificity), and 311 for RP lipidomics (88.1% specificity). Notably, few metabolites were shared between the two extraction phases (metabolomics/lipidomics) and between the three LC- methods, highlighting the complementarity nature and added value of integrating these three analytical approaches on the two extraction phases (Details in SI 1).

Using NPClassifier (Kim et al., 2021), the major classes and subclasses of compounds were identified based on their biosynthetic pathways. Noteworthy compound classes identified in the metabolomics-RP analysis included shikimates and phenylpropanoids (e.g. stilbenoids, flavonoids, coumarins, etc.), terpenoids (mono-, di-, tri-terpenoids, etc.), and fatty acids with fatty acyls being predominant. For metabolomics-HILIC, key compound classes included shikimates and phenylpropanoids (i.e. flavonoids, stilbenoids, coumarins, lignans, etc.), amino acids and peptides, and alkaloids. In lipidomics-RP, fatty acids (e.g. glycerolipids, glycerophosphilipids, sphingolipids, etc.) and terpenoids (e.g. steroids, triterpenoids) were the primary compounds identified (Fig. 1B-D). These major classes are consistent with the polarity of the two extracted phases and chromatographic separations methods. The integration of these three types of LC-MS analytical approaches represents an innovative strategy to increase metabolome coverage. To the best of our knowledge, this type of comprehensive analysis has not yet been applied to grapevine.

### Integrative statistical approach for key grapevine biomarkers in esca disease resistance

**Fig. 2:**
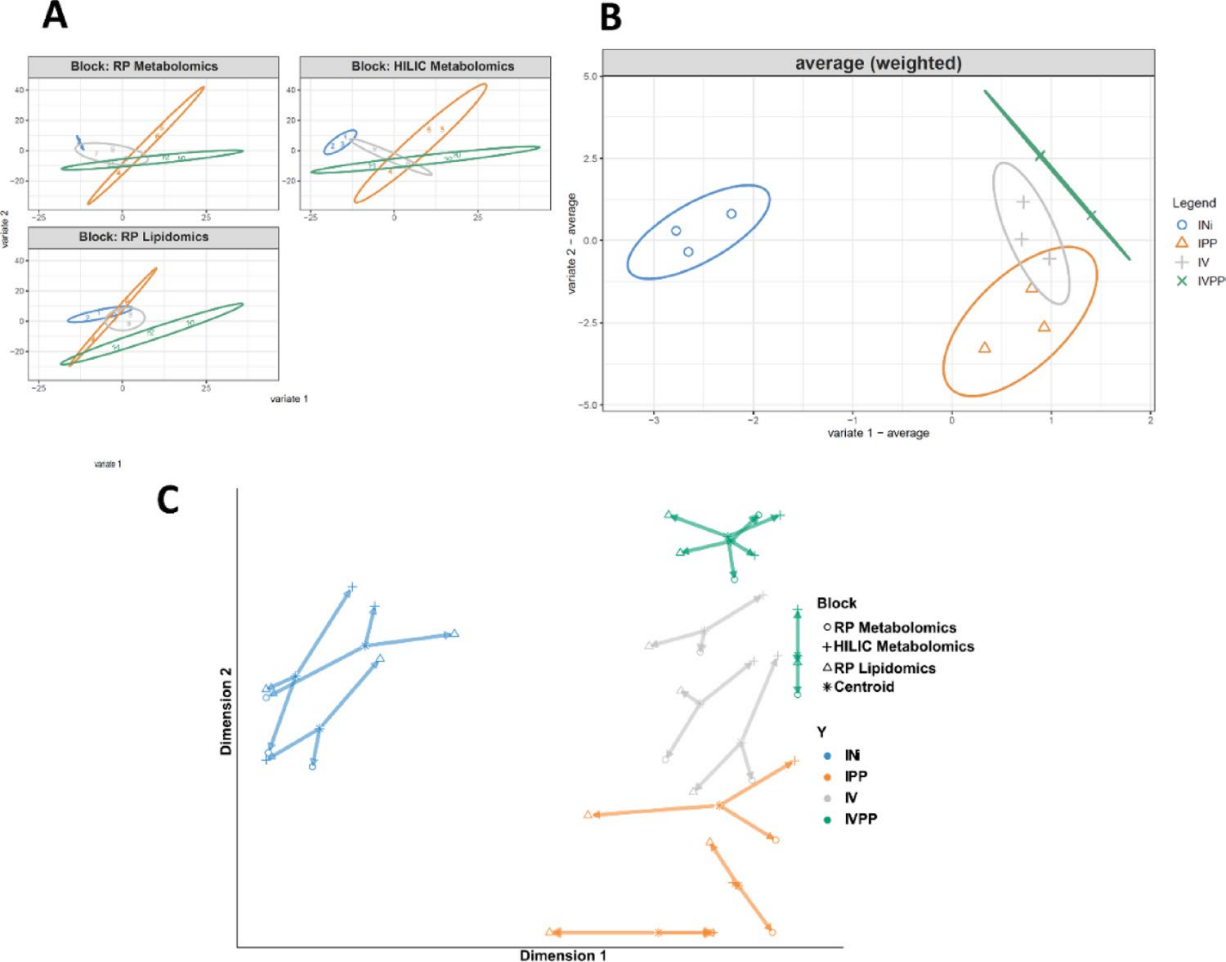
Multivariate analysis (PCA and arrow plot) illustrating the integration of the three analytical approaches. **A** PCA for each analytical method. **B** Average PCA for all three analytical methods using DIABLO analysis (components 1and 2). **C** Arrow plot in two dimensions for all samples analyzed with three LCMS analysis methods. *In 2-A*,* the numbers correspond to the columns of the analysis matrices*

To identify metabolic signatures associated with fungal infection and biocontrol, we employed a multi-block statistical approach. Initial PCA of the individual datasets showed only modest separation of the experimental groups, except for the lipidomics data, which began to discriminate the treatments (Fig. 2A). However, when the three datasets were integrated using DIABLO, a clear separation emerged (Fig. 2B). The integrated PCA showed that samples infected with pathogenic fungi (IPP and IVPP) and the biocontrol agent (IV) were separated from the control (INi) along the first component. An arrow plot analysis confirmed the strong correlation between the data blocks and the robustness of the sample grouping (Fig. 2C).

**Fig. 3:**
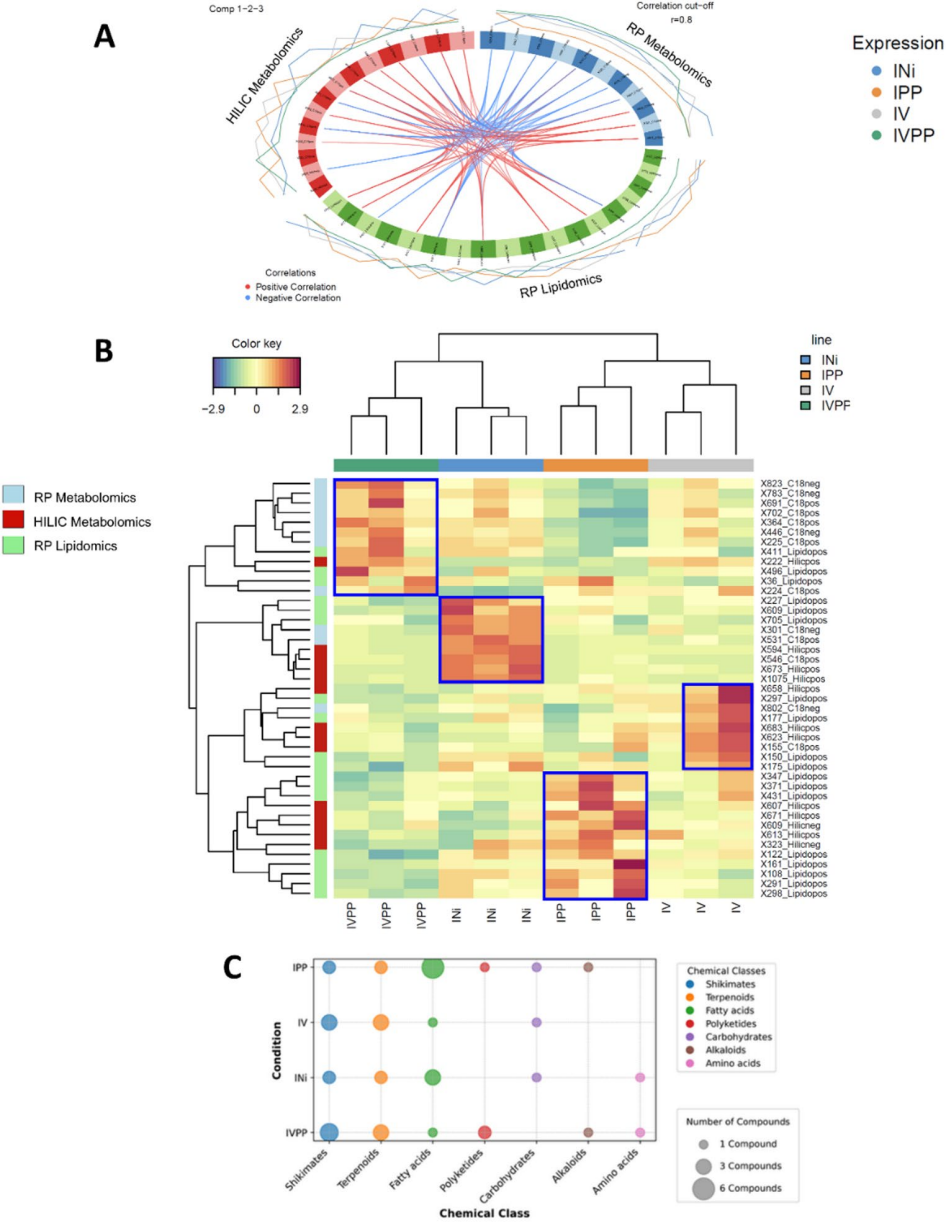
**A** Statistical analyses to identify the distribution of 43 discriminating biomarkers by class of samples: circosplot, **B** CIM, **C** NP classifier pathways for the top biomarkers by class. The figure illustrates the number of metabolites in each class

To pinpoint the most discriminating variables, we generated a circosplot and a Clustered Image Map (CIM) from the DIABLO model (Fig. 3A, B). The circosplot revealed a strong positive correlation between the HILIC and lipidomics datasets, while the RP dataset showed a weaker, negative correlation, further emphasizing their chemical orthogonality. The CIM highlighted the top 50 discriminating biomarkers across the four treatment groups, with their identities detailed in Table 1. Of the 43 most discriminating features, the lipidomics dataset contributed the largest share (18 features), followed by RP metabolomics (13 features) and HILIC metabolomics (12 features). These biomarkers were mapped to diverse biosynthetic pathways, including shikimates/phenylpropanoids (11), terpenoids (10), fatty acids (9), and others (Fig. 3C), underscoring the systemic nature of the plant’s response.

**Table 1:** Features identified by method and class

Feature ID	m/z [M + H]^+^	RT	Formula	Annotation	Annotation level	Chemical class/pathway
				**Class INi**		
				*C18*		
301_neg	297.24329	12.02	C_18_H_34_O_3_	(R)-(E)−12-Hydroxy-9-octadecenoic acid	Level3 = InSilico-Generic	Hydroxy fatty acids|Other Octadecanoids/fatty acids
531_pos	311.16385	12.47	C_20_H_22_O_3_	7,4’-Dihydroxy-3’-prenylflavan	Level3 = InSilico-Generic	Flavans/shikimates and phenylpropanoids
546_pos	318.12939	0.91	C_12_H_19_N_3_O_7_	N-(1-Deoxy-1-fructosyl)histidine	Level3 = InSilico-Generic	Carboxylic acids and derivatives/carbohydrates
				*HILIC*		
594_pos	333.14639	1.26	C_20_H_22_O_3_^§^	Obtusifolin	Level3 = InSilico-Generic	Coumarins/shikimates and phenylpropanoids
673_pos	369.38419	2.19	C_23_H_48_N_2_O	N-[2-[heptadecyl(methyl)amino]ethyl]-N-methylacetamide	Level3 = InSilico-Generic	N-acyl amines/fatty acids
1075_pos	766.46399	1.86	C_39_H_70_NO_10_P^§^	PS(15:0/18:3(6Z,9Z,12Z))	Level3 = InSilico-Generic	Glycerophosphoserines/fatty acids
				*LIPIDOMIC*		
227_pos	568.42773	4.36	C_40_H_56_O_2_	Zeaxanthin	Level4 = MSMSanalog	Carotenoids/terpenoids
609_pos	921.25177	0.83	C_24_H_20_N_4_O_4_S^‡^	*3-(3-{[3-(4-methylbenzenesulfonamido)quinoxalin-2-yl]amino}phenyl)prop-2-enoic acid*	Level3 = InSilico-Generic	UNK/amino acids
705_pos	1376.22034	17.59	C_47_H_80_O_2_^§^	20:2 Cholesteryl ester	Level3 = InSilico-Generic	Cholestane steroids/terpenoids
				**Class IV**		
				*C18*		
802_neg	571.28851	11.64	C_32_H_44_O_9_	(3beta,5beta,11alpha)−3-[(7-Carboxy-1-oxoheptyl)oxy]−11,14-dihydroxy-12-oxo-bufa-20,22-dienolide	Level3 = InSilico-Generic	Bufadienolides/terpenoids
155_pos	167.07028	3.59	C_9_H_10_O	4-(3-hydroxy-1-propen-1-yl)−1,2-benzenediol	Level3 = InSilico-Generic	Cinnamic acids and derivatives/shikimates and phenylpropanoids
				*HILIC*		
658_pos	363.12646	3.04	C_15_H_22_O_10_	1-O-methyl-3,4,5,6-tetraacetyl-epi-inositol	Level3 = InSilico-Generic	UNK/carbohydrates
683_pos	373.147	2.5	C_17_H_26_O_10_^†^	3’,4’-Di-Me ether, 2-O-D-glucopyranoside	Level3 = InSilico-Generic	Glycoside/shikimates and phenylpropanoids
623_pos	345.11588	3.71	C_20_H_18_O_4_^§^	4-oxo-3-phenyl-6-propyl-4 H-chromen-7-yl acetate	Level4 = MSMSanalog	Isoflavones/shikimates and phenylpropanoids
				*LIPIDOMIC*		
297_pos	663.45319	11.32	C_20_H_32_O_3_^§^	11(R)-HETE	Level3 = InSilico-Generic	Hydroxy-hydroperoxyeicosatetraenoic acids/fatty acids
177_pos	475.37842	8.49	C_30_H_50_O_4_	(22R)-acetoxy-(24)-ergosta-5-en-3,25-diol	Level3 = InSilico-Generic	Cholestane steroids|Ergostane steroids/terpenoids
150_pos	429.37262	5.61		Unknown		
175_pos	469.36508	4.72	C_31_H_48_O_3_	25-hydroxy-1alpha-hydroxymethyl-26,27-dimethyl-24a-homo-22,23,24,24a-tetradehydrovitamin D3	Level3 = InSilico-Generic	Vitamin D3 and derivatives/terpenoids
				**Class IPP**		
				*HILIC*		
607_pos	338.34171	1.33	C_22_H_43_NO	13-Docosenamide	Level3 = InSilico-Generic	Fatty acyls/fatty acids
671_pos	369.11572	1.58	C_15_H_22_O_9_^§^	(1xi,2xi)−1-(4-Hydroxyphenyl)−1,2,3-propanetriol 2-O-beta-D-glucopyranoside	Level3 = InSilico-Generic	Phenylethanoids/shikimates and phenylpropanoids
609_neg	389.12424	2.27	C_20_H_22_O_8_	Resveratrol 3-beta-mono-D-glucoside (trans-piceid)	Level2 = MSMSmatch	Monomeric stilbenes/shikimates and phenylpropanoids
613_pos	341.08401	2.54	C_15_H_16_O_9_	5-O-D-Glucopyranoside	Level3 = InSilico-Generic	Coumarin/polyketides
323_neg	215.03253	5.18	C_6_H_12_O_6_^¶^	Inositol	Level3 = InSilico-Biosource	Cyclitols/carbohydrates
				*LIPIDOMIC*		
347_pos	707.49127	11.1	C_37_H_71_O_10_P	PA(i-16:0/18:1(12Z)−2OH(9,10))	Level3 = InSilico-Generic	Glycerophosphates/fatty acids
371_pos	722.52692	11.13	C_41_H_68_O_9_^&^	MGDG O-28:6_4:0	Level4 = MSMSanalog	Glycosyldiacylglycerols/fatty acids
431_pos	784.52637	11.12	C_48_H_66_N_2_O_6_^&^	17-[4-[3-[2-(3-aminophenyl)cyclopentyl]−3-methyloxiran-2-yl]−4-hydroxybutan-2-yl]−6-hydroxy-2-[3-hydroxy-5-(methylaminomethyl)phenyl]−4,4,8,10,14-pentamethyl-2,5,6,7,9,11,12,15-octahydro-1 H-cyclopenta[a]phenanthrene-3,16-dione	Level3 = InSilico-Generic	Triterpenoids/terpenoids
122_pos	395.36731	4.84	C_29_H_48_O^†^	24-Methylenepollinastanol	Level3 = InSilico-Generic	Cholestane steroids/terpenoids
161_pos	445.1199	5.61	C_20_H_20_N_4_O_6_S	(3R,5 S)−1-[(4-methanesulfonylphenyl)methyl]−5-[3-(3-nitrophenyl)−1,2,4-oxadiazol-5-yl]pyrrolidin-3-ol	Level3 = InSilico-Generic	Azoles/alkaloids
108_pos	371.24054	3.45	C_20_H_34_O_6_	(E)−5,8-dihydroxy-8-(4-hydroxy-5-((Z)-oct-2-en-1-yl)tetrahydrofuran-2-yl)oct-6-enoic acid (delta6-9-IsoF)	Level3 = InSilico-Generic	Isofurans-eicosanoids/fatty acids
291_pos	663.45282	11.51	C_20_H_32_O_3_^@^	13-HETE	Level3 = InSilico-Generic	Hydroxy-hydroperoxyeicosatetraenoic acids/Fatty acids
298_pos	663.45337	13.55	C_20_H_32_O_3_^@^	16(R)-HETE	Level3 = InSilico-Generic	Hydroxy-hydroperoxyeicosatetraenoic acids/fatty acids
				**Class IVPP**		
				*C18*		
823_neg	583.21844	7.59	C_31_H_36_O_11_	[13,18-Diacetyloxy-5-hydroxy-10-(2-hydroxypropan-2-yl)−7-methyl-4-oxo-3,15-dioxapentacyclo[10.6.0.01,5.06,10.013,16]octadec-6-en-11-yl] benzoate	Level3 = InSilico-Generic	Abeotaxane diterpenoids/terpenoids
783_neg	555.22327	6.97	C_30_H_36_O_10_	Hainangranatumin C	Level3 = InSilico-Generic	Limonoids/terpenoids
691_pos	397.16483	6.95	C_21_H_26_O_6_^§^	Strobilurin I	Level3 = InSilico-Generic	Strobilurins and derivatives/polyketides
702_pos	401.15958	7.68	C_22_H_24_O_7_	6-Acetylteuscordin	Level3 = InSilico-Generic	Colensane and clerodane diterpenoids/terpenoids
364_pos	249.11227	6.89	C_7_H_8_O_2_^‡^	2-Methylresorcinol	Level3 = InSilico-Generic	Resorcinol/shikimates and phenylpropanoids
446_neg	361.16577	6.81	C_20_H_26_O_6_	Secoisolariciresinol	Level2 = MSMSmatch	Dibenzylbutane lignans/shikimates and phenylpropanoids
225_pos	194.05943	7.11	C_10_H_10_O_4_^†^	Ferulate	Level4 = MSMSanalog	Cinnamic acids and derivatives/shikimates and phenylpropanoids
224_pos	193.08583	5.73	C_11_H_14_O_4_^¥^	4-hydroxy-3,5-dimethoxy-cinnamyl alcohol (Sinapyl alcohol)	Level2 = MSMSmatch	Cinnamic acids and derivatives/shikimates and phenylpropanoids
				*HILIC*		
222_pos	178.10756	4.91	C_7_H_15_NO_4_	2-amino-4,5-dihydroxy-3,4-dimethylpentanoic acid	Level3 = InSilico-Generic	Aminoacids/amino acids and peptides
				*LIPIDOMIC*		
411_pos	764.61896	13.43	C_42_H_83_O_8_P^&^	PMeOH 38:0	Level4 = MSMSanalog	Glycerophosphates/fatty acids
496_pos	846.75378	16.83	C_53_H_96_O_6_^&^	[1-[5-(1-hydroxytridecyl)oxolan-2-yl]−13-(2-methyl-5-oxo-2 H-furan-4-yl)tridecyl] octadec-9-enoate	Level3 = InSilico-Generic	Acetogenins/polyketides
36_pos	188.07071	0.82	C_11_H_11_NO_3_^†^	DL-Indole-3-lactic acid	Level2 = MSMSmatch	Simple indole alkaloids/alkaloid

Pos = positive ionization [M + H]^+^; ^†^= [M + H-2H_2_O]^+^; ^¥^= M; ^‡^ = [2 M + H]^+^; ^§^ = [M + Na]^+^; ^¶^ = [M + Cl]^−^; ^&^ = [M+NH4]^+^; ^@^ = [2 M + Na]^+^. Neg = negative ionization [M-H]^−^. Annotation level: 1 = internal database; 2 = match on MSMS spectra (low external data); 3 = VitiCyc, genus, family, generic; 4 = MS/MS analogues. See Supplementary Information (SI 2) for full structural annotations generated with NPClassyfire

### Oxylipin signaling is a central component of the grapevine defense response

Our integrative analysis revealed that fatty acid metabolism was profoundly altered in response to pathogenic infection. The IPP group (co-infected with *P. chlamydospora* and *P. minimum*) showed a dramatic increase in the abundance of specific fatty acids compared to the control INi group (Fig. 3C). This finding suggests that lipids play a dual role: a structural role in healthy tissue (e.g., phosphatidylcholines in the INi group) and a signaling or defensive role in infected tissue. For the IVPP class (Injured/P. *chlamydospora* + *P. minimum* + Vintec^®^), 9 distinct features were identified in the metabolomic analysis (1 in HILIC and 8 in RP) and 3 in the lipidomic analysis. In the IV class (Injured/Vintec^®^), 5 metabolomic features (3 in HILIC and 2 in RP) and 4 lipidomic features were detected. Among the 13 features identified for IPP class (Injured/*P. chlamydospora* + *P. minimum*), including 5 for HILIC and 8 for lipidomics, fatty acids, were notably more abundant, while other classes exhibited more heterogenous compound profiles. In the control class (INi, Injured/Not inoculated), 3 compounds were identified across each analytical method.

The marked influence of fatty acids in IPP class compared to the control group INi (Fig. 3C), may be attributed to fungal inoculation. Using our analytical methodologies, particularly lipidomics, fatty acids appear to play a dual role: a structural role, as seen with phosphatidylcholines (INi), which are major glycerolipid components of most membranes, and the signalizations or defensive role, as observed with oxylipins (IPP and IV).

Advances in plant lipidome research have provided detailed insights into its biosynthesis, regulation, adaptation, remodeling, functions, and interactions (Tenenboim et al., 2016). The integration of HILIC with lipidomic approaches can significantly improve lipid class identification and potentially enable the discovery of novel lipid classes. To date there are few lipidomic studies on the grapevines and the cause-effect biochemical relationship of esca disease. Goufo and Cortez (2020), reported that a lipidomic analysis of *V. vinifera* L. leaves, was conducted to assess how lipid membrane remodeling relates to the emergence and progression of Esca foliar symptoms. Changes in the lipid composition of leaves have been primarily reported for galactolipids, which are associated with chemical signaling and plant defense mechanisms (Goufo & Cortez, 2020).

In this study, the role of fatty acids, particularly oxylipins, is explored as potential alarmins in response to trunk infection by esca disease. Oxylipins constitute a large family of oxidized fatty acids and metabolites derived therefrom. These bioactive lipids are abundant in mammals as well as in nonmammals, including flowering plants, mosses, algae, bacteria and fungi (de León et al., 2015; Pohl & Kock, 2014). In plants, they serve as signal molecules regulating developmental processes such as pollen formation, and mediate responses to biotic and abiotic stresses such as herbivore or pathogen attack and desiccation (Deboever et al., 2020).

The literature extensively describes oxylipins as key signaling molecules in plant defense, with numerous studies highlighting their strong interplay with phytohormones and their involvement in antimicrobial activity against pathogens (de León et al., 2015; Deboever et al., 2020; Mosblech et al., 2009; Prost et al., 2005). Jasmonic acids (JAs) are key oxylipin-derived phytohormones involved in plant defense signaling, particularly in response to biotic stress and wounding. In grapevine, they regulate defense-related gene expression and interact with other phytohormones such as salicylic acid and ethylene. While JAs were not detected in our current dataset, this likely reflects biological and analytical limitations such as their rapid, transient accumulation and low ionization efficiency in complex matrices rather than their absence from the grapevine defense response. In our study, the response to esca infection is driven by the balance between pro- and anti-inflammatory signaling mediated by oxylipins, principally Hydroxyeicosatetraenoic acids (HETEs). This is evidenced by the production of two oxylipins, 13-HETE and 16(R)-HETE, following fungal inoculation (IPP); and one oxylipin, 11(R)-HETE, during biocontrol inoculation (IV). The predominant pathway for HETE formation involves the peroxidase-catalyzed reduction of hydroperoxyeicosatetraenoic acids (HpETEs), which are generated by various lipoxygenases (LOX). These enzymes are widely distributed across animals, plants, fungi, and certain bacteria (Beccaccioli et al., 2022). Although mammalian LOX are classified based on the position at which they introduce a molecule of oxygen into arachidonic acid (AA), AA is not necessarily their preferred substrate (Powell & Rokach, 2014). In another fungal species, 18-HETE is a specific metabolite produced by *Malassezia*, primarily biosynthesized by *M. furfur* and associated with its virulence on human skin (Ambaw et al., 2021). In *Dipodascus uninucleatus*, the addition of arachidonic acid leads to the predominant production of 3-HETE (van Dyk et al., 1991). It is worth noting that while the biphasic extraction and LC-MS methods employed here provide broad metabolome coverage, particularly for oxidized fatty acids, our lipidomic approach may not fully capture the complete lipidome. Alternative extraction protocols, such as chloroform-based solvent systems, are known to enhance lipid recovery, and complementary analytical platforms, including GC-MS or direct-infusion LC-MS, could further refine the characterization of lipid classes involved in plant defense signaling.

Other non-lipid molecules involved in the attack-response interaction were identified in classes IV and IVPP. Shikimates and phenylpropanoids (three molecules in each class) were detected as plant defense compounds, primarily isoflavones, lignans, and cinnamic acid derivatives. Additionally, terpenes, which serve as plant signaling molecules and are likely induced by infection, were identified (three molecules in each class). These included bufadienolides, cholestane steroids, abietane, limonoids, and clerodane derivatives.

While our multiplexed LC-MS approach significantly expands the known metabolome and lipidome of grapevine wood, we recognize that certain classes of defense-related molecules remain outside the scope of this study. Volatile organic compounds (VOCs), such as terpenes and green leaf volatiles, represent a crucial ‘first line’ of chemical signaling in plant-pathogen interactions that require specialized headspace sampling and GC-MS platforms for detection. Similarly, the transient and low-abundance nature of certain phytohormones often necessitates targeted, high-sensitivity MRM (Multiple Reaction Monitoring) methods to overcome matrix effects and ionization limitations. Finally, a truly comprehensive view of the grapevine’s systemic response to *esca* would benefit from the future integration of macromolecular analyses, including proteomics to identify pathogenesis-related (PR) proteins and glycomics to characterize structural cell-wall modifications.

## Conclusions

Exploring an organism’s complete metabolome requires a combination of efficient extraction, advanced analytical methodologies, and robust data integration. The multiplexed approach developed in this study, which pairs biphasic extraction with three orthogonal LC-MS methods, provides a powerful framework for achieving comprehensive metabolome coverage in complex plant tissues. By applying this strategy to grapevine wood, we significantly increased the number of detected metabolites and uncovered chemical responses to fungal infection that were previously invisible.

Our multi-block data analysis successfully deciphered the contribution of each analytical method and facilitated the identification of a wide array of discriminating biomarkers. The most significant finding was the identification of oxidized fatty acids, specifically HETEs, as putative signaling molecules in the *Vitis vinifera* defense response to Esca pathogens. This discovery highlights the critical importance of including lipidomics in studies of plant-pathogen interactions.

While our approach provides broad coverage, future work could incorporate complementary analytical platforms (e.g., GC-MS) or alternative extraction protocols to further refine the characterization of specific lipid classes.

## Supplementary Information

Below is the link to the electronic supplementary material.

Supplementary Material 1

Supplementary Material 2

## Data Availability

The raw data from the LCMS were uploaded to zenodo (10.5281/zenodo.17095265).
