# Supplementary material for: Multiplexed LC-MS analysis reveals novel insights into grapevine defense mechanisms by expanding metabolome coverage
Source: Metabolomics. 2026 Mar 7;22(2):35. doi: 10.1007/s11306-026-02410-y (PMC12967536; doi:10.1007/s11306-026-02410-y)
Supplement: Supplementary file 1 — Supplementary Material 1 [file 11306_2026_2410_MOESM1_ESM.docx]

Multiplexed LC-MS analysis reveals novel insights into grapevine defense mechanisms by expanding metabolome coverage

Pedro G. Vásquez-Ocmín^a,b*^, Amelie Pérez^a,b,c^, Ana Romeo-Oliván^d^, Virginie Puech-Pages^a,b,c^, Sylvie Fournier^a,b,c^, Bernard Dumas^a^, Alban Jacques^d^, Guillaume Marti^a,b,c*^

^a^Laboratoire de Recherche en Sciences Végétales, Université de Toulouse, CNRS, Toulouse INP, Toulouse, France

^b^Metatoul-AgromiX Platform, LRSV, Université de Toulouse, CNRS, Toulouse INP, Toulouse, France

^c^MetaboHUB-MetaToul, National Infrastructure of Metabolomics and Fluxomics, Toulouse, France

^d^Université de Toulouse, Ecole d’Ingénieurs de PURPAN, PPGV, Toulouse, France

*[vasco2224@gmail.com](mailto:vasco2224@gmail.com) / [guillaume.marti@utoulouse.fr](mailto:guillaume.marti@utoulouse.fr)

**SI 2. Full structural annotations generated with NPClassyfire**

**Table 1.** Features identified by method and class. Pos = positive ionization [M+H]^+^; ^†^= [M+H-2H_2_O]^+^; ^¥^= M; ^‡^ = [2M+ H]^+^ ; ^§^ = [M+ Na]^+^; ^¶^ = [M+Cl]^-^; ^&^ = [M+NH4]^+^; ^@^ = [2M+Na]^+^. Neg = negative ionization [M-H]^-^. Annotation level: 1 = internal database; 2 = match on MSMS spectra (low external data); 3 = VitiCyc, genus, family, generic; 4 = MS/MS analogues. See Supplementary Information (SI 2) for full structural annotations generated with NPClassyfire.

| **Feature ID** | ***m/z* [M+H]^+^** | **RT** | **Formula** | **Annotation** | **Structure**  **(from NP classyfire)** | **Annotation level** | **Chemical class/Pathway** |
| --- | --- | --- | --- | --- | --- | --- | --- |
|  |  |  |  | **Class INi** |  |  |  |
|  |  |  |  | *C18* |  |  |  |
| 301_neg | 297.24329 | 12.02 | C_18_H_34_O_3_ | (R)-(E)-12-Hydroxy-9-octadecenoic acid | 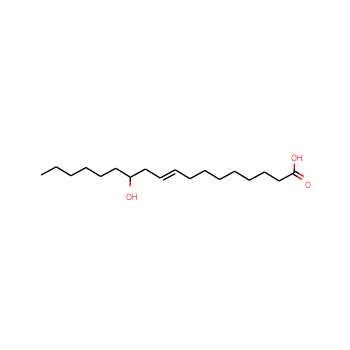 | Level3=InSilico-Generic | *Hydroxy fatty acids\|Other Octadecanoids/* *Fatty acids* |
| 531_pos | 311.16385 | 12.47 | C_20_H_22_O_3_ | 7,4'-Dihydroxy-3'-prenylflavan | 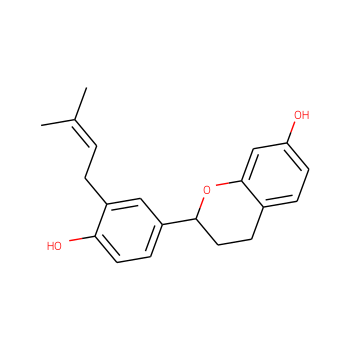 | Level3=InSilico-Generic | *Flavans / Shikimates and Phenylpropanoids* |
| 546_pos | 318.12939 | 0.91 | C_12_H_19_N_3_O_7_ | N-(1-Deoxy-1-fructosyl)histidine | 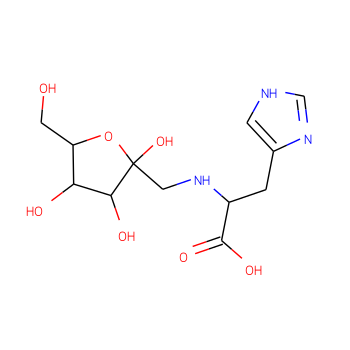 | Level3=InSilico-Generic | *Carboxylic acids and derivatives /Carbohydrates* |
|  |  |  |  | *HILIC* |  |  |  |
| 594_pos | 333.14639 | 1.26 | C_20_H_22_O_3_^§^ | Obtusifolin | 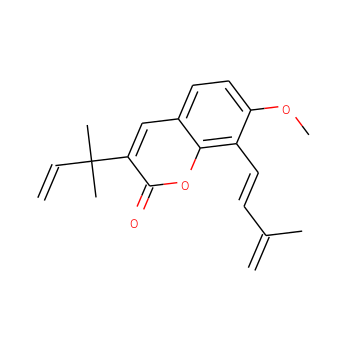 | Level3=InSilico-Generic | *Coumarins / Shikimates and Phenylpropanoids* |
| 673_pos | 369.38419 | 2.19 | C_23_H_48_N_2_O | N-[2-[heptadecyl(methyl)amino]ethyl]-N-methylacetamide | 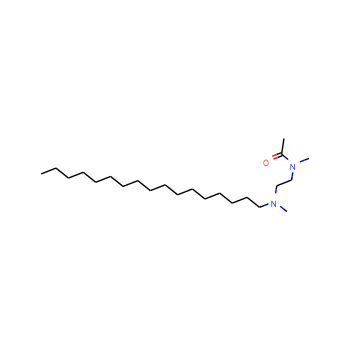 | Level3=InSilico-Generic | *N-acyl amines /Fatty acids* |
| 1075_pos | 766.46399 | 1.86 | C_39_H_70_NO_10_P^§^ | PS(15:0/18:3(6Z,9Z,12Z)) | 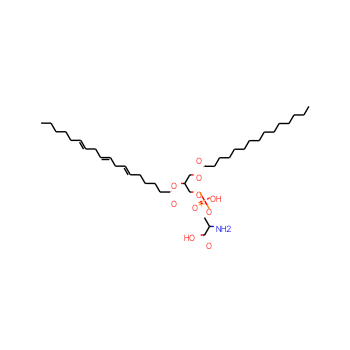 | Level3=InSilico-Generic | *Glycerophosphoserines / Fatty acids* |
|  |  |  |  | *LIPIDOMIC* |  |  |  |
| 227_pos | 568.42773 | 4.36 | C_40_H_56_O_2_ | *Zeaxanthin* | 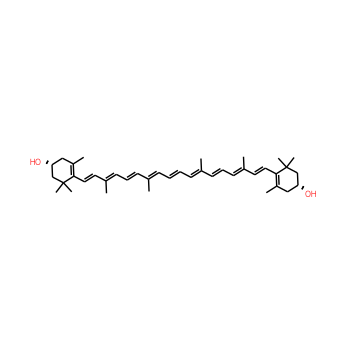 | Level4=MSMSanalog | *Carotenoids / Terpenoids* |
| 609_pos | 921.25177 | 0.83 | C_24_H_20_N_4_O_4_S^‡^ | *3-(3-{[3-(4-methylbenzenesulfonamido)quinoxalin-2-yl]amino}phenyl)prop-2-enoic acid* | 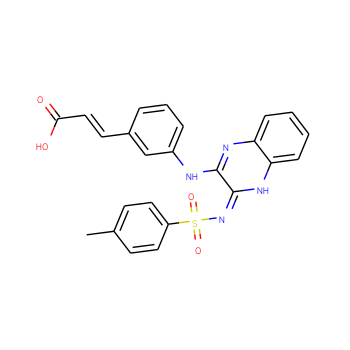 | Level3=InSilico-Generic | *UNK/Amino acids* |
| 705_pos | 1376.22034 | 17.59 | C_47_H_80_O_2_^§^ | *20:2 Cholesteryl ester* | 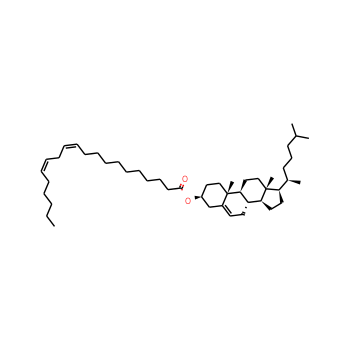 | Level3=InSilico-Generic | *Cholestane steroids / Terpenoids* |
|  |  |  |  | **Class IV** |  |  |  |
|  |  |  |  | *C18* |  |  |  |
| 802_neg | 571.28851 | 11.64 | C_32_H_44_O_9_ | (3beta,5beta,11alpha)-3-[(7-Carboxy-1-oxoheptyl)oxy]-11,14-dihydroxy-12-oxo-bufa-20,22-dienolide | 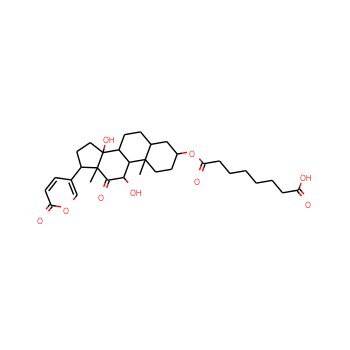 | Level3=InSilico-Generic | *Bufadienolides / Terpenoids* |
| 155_pos | 167.07028 | 3.59 | C_9_H_10_O | 4-(3-hydroxy-1-propen-1-yl)-1,2-benzenediol | 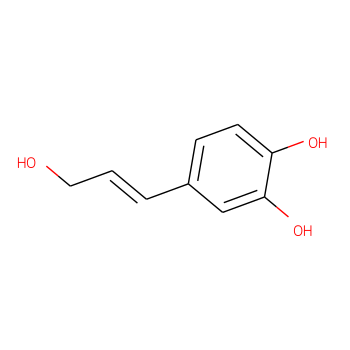 | Level3=InSilico-Generic | *Cinnamic acids and derivatives / Shikimates and Phenylpropanoids* |
|  |  |  |  | *HILIC* |  |  |  |
| 658_pos | 363.12646 | 3.04 | C_15_H_22_O_10_ | 1-O-methyl-3,4,5,6-tetraacetyl-epi-inositol | 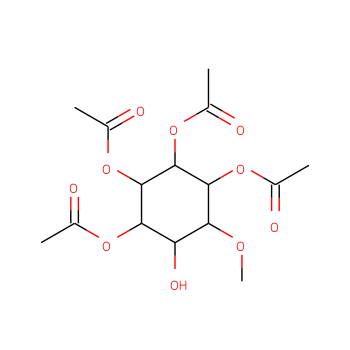 | Level3=InSilico-Generic | *UNK / Carbohydrates* |
| 683_pos | 373.147 | 2.5 | C_17_H_26_O_10_^†^ | 3',4'-Di-Me ether, 2-O-?-D-glucopyranoside | 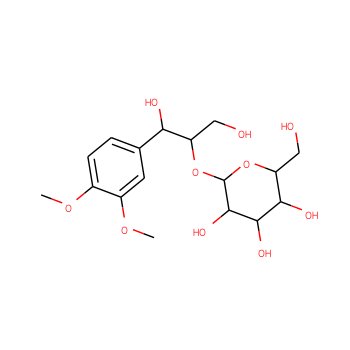 | Level3=InSilico-Generic | *Glycoside / Shikimates and Phenylpropanoids* |
| 623_pos | 345.11588 | 3.71 | C_20_H_18_O_4_^§^ | 4-oxo-3-phenyl-6-propyl-4H-chromen-7-yl acetate | 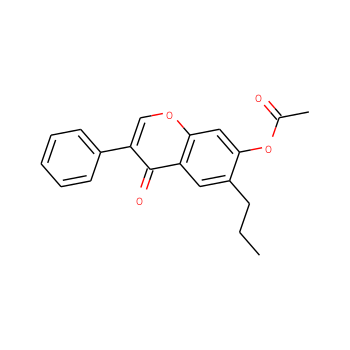 | Level4=MSMSanalog | *Isoflavones / Shikimates and Phenylpropanoids* |
|  |  |  |  | *LIPIDOMIC* |  |  |  |
| 297_pos | 663.45319 | 11.32 | C_20_H_32_O_3_^§^ | 11(R)-HETE | 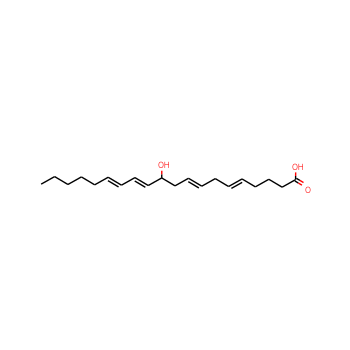 | Level3=InSilico-Generic | *Hydroxy-hydroperoxyeicosatetraenoic acids / Fatty acids* |
| 177_pos | 475.37842 | 8.49 | C_30_H_50_O_4_ | (22R)-acetoxy-(24?)-ergosta-5-en-3?,25-diol | 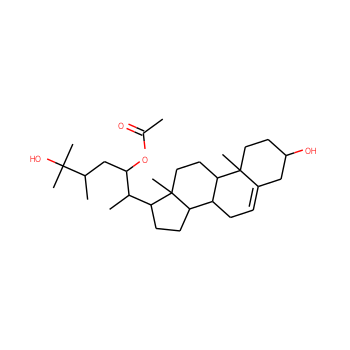 | Level3=InSilico-Generic | *Cholestane steroids\|Ergostane steroids / Terpenoids* |
| 150_pos | 429.37262 | 5.61 |  | Unknown |  |  |  |
| 175_pos | 469.36508 | 4.72 | C_31_H_48_O_3_ | 25-hydroxy-1alpha-hydroxymethyl-26,27-dimethyl-24a-homo-22,23,24,24a-tetradehydrovitamin D3 | 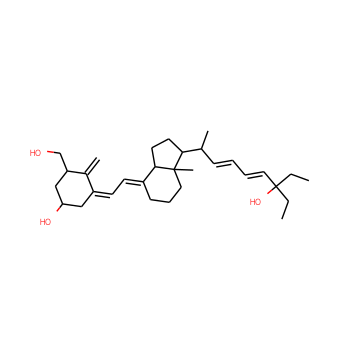 | Level3=InSilico-Generic | *Vitamin D3 and derivatives/Terpenoids* |
|  |  |  |  | **Class IPP** |  |  |  |
|  |  |  |  | *HILIC* |  |  |  |
| 607_pos | 338.34171 | 1.33 | C_22_H_43_NO | 13-Docosenamide | 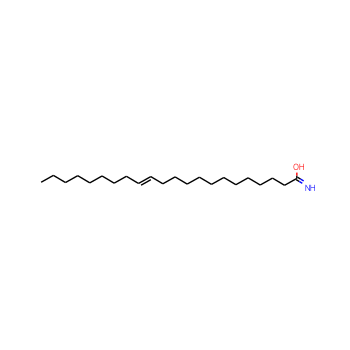 | Level3=InSilico-Generic | *Fatty acyls / Fatty acids* |
| 671_pos | 369.11572 | 1.58 | C_15_H_22_O_9_^§^ | (1xi,2xi)-1-(4-Hydroxyphenyl)-1,2,3-propanetriol 2-O-beta-D-glucopyranoside | 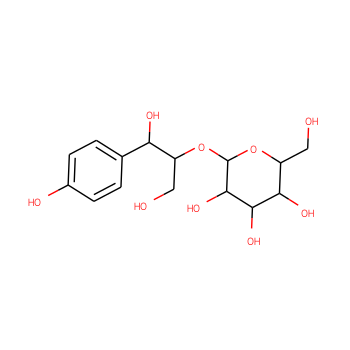 | Level3=InSilico-Generic | *Phenylethanoids / Shikimates and Phenylpropanoids* |
| 609_neg | 389.12424 | 2.27 | C_20_H_22_O_8_ | Resveratrol 3-beta-mono-D-glucoside (trans-piceid) | 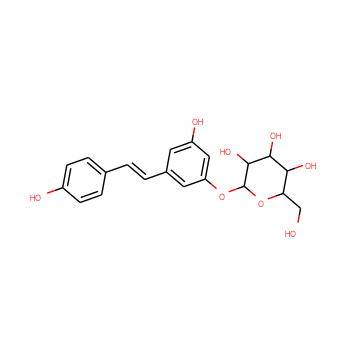 | Level2=MSMSmatch | *Monomeric stilbenes /Shikimates and Phenylpropanoids* |
| 613_pos | 341.08401 | 2.54 | C_15_H_16_O_9_ | 5-O-D-Glucopyranoside | 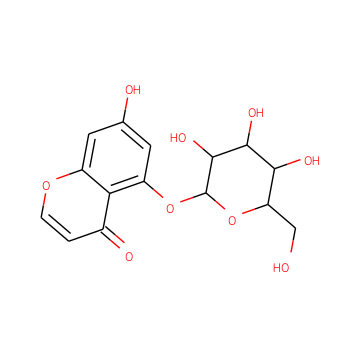 | Level3=InSilico-Generic | *Coumarin / Polyketides* |
| 323_neg | 215.03253 | 5.18 | C_6_H_12_O_6_^¶^ | Inositol | 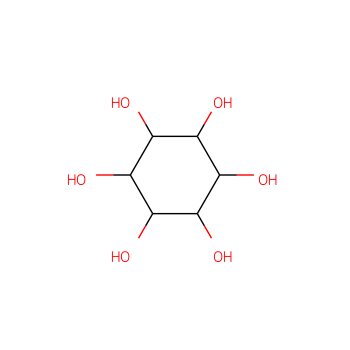 | Level3=InSilico-Biosource | *Cyclitols / Carbohydrates* |
|  |  |  |  | *LIPIDOMIC* |  |  |  |
| 347_pos | 707.49127 | 11.1 | C_37_H_71_O_10_P | PA(i-16:0/18:1(12Z)-2OH(9,10)) | 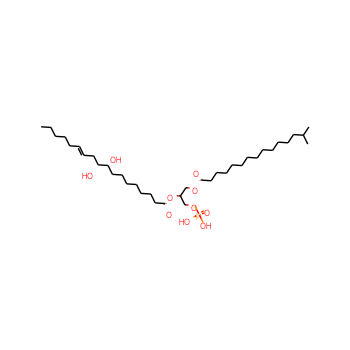 | Level3=InSilico-Generic | *Glycerophosphates /Fatty acids* |
| 371_pos | 722.52692 | 11.13 | C_41_H_68_O_9_^&^ | MGDG O-28:6_4:0 | 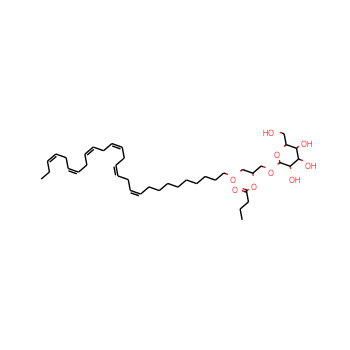 | Level4=MSMSanalog | *Glycosyldiacylglycerols/Fatty acids* |
| 431_pos | 784.52637 | 11.12 | C_48_H_66_N_2_O_6_^&^ | 17-[4-[3-[2-(3-aminophenyl)cyclopentyl]-3-methyloxiran-2-yl]-4-hydroxybutan-2-yl]-6-hydroxy-2-[3-hydroxy-5-(methylaminomethyl)phenyl]-4,4,8,10,14-pentamethyl-2,5,6,7,9,11,12,15-octahydro-1H-cyclopenta[a]phenanthrene-3,16-dione | 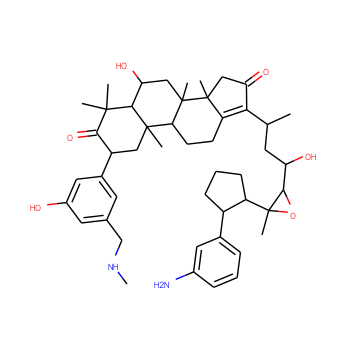 | Level3=InSilico-Generic | *Triterpenoids / Terpenoids* |
| 122_pos | 395.36731 | 4.84 | C_29_H_48_O^†^ | 24-Methylenepollinastanol | 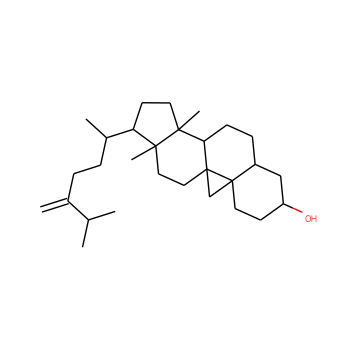 | Level3=InSilico-Generic | *Cholestane steroids / Terpenoids* |
| 161_pos | 445.1199 | 5.61 | C_20_H_20_N_4_O_6_S | (3R,5S)-1-[(4-methanesulfonylphenyl)methyl]-5-[3-(3-nitrophenyl)-1,2,4-oxadiazol-5-yl]pyrrolidin-3-ol | 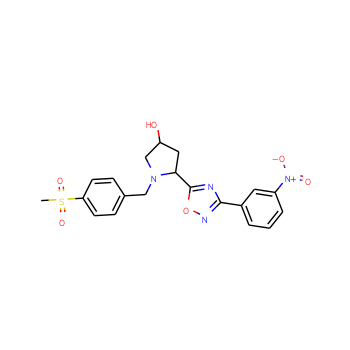 | Level3=InSilico-Generic | *Azoles /Alkaloids* |
| 108_pos | 371.24054 | 3.45 | C_20_H_34_O_6_ | (E)-5,8-dihydroxy-8-(4-hydroxy-5-((Z)-oct-2-en-1-yl)tetrahydrofuran-2-yl)oct-6-enoic acid (delta6-9-IsoF) | 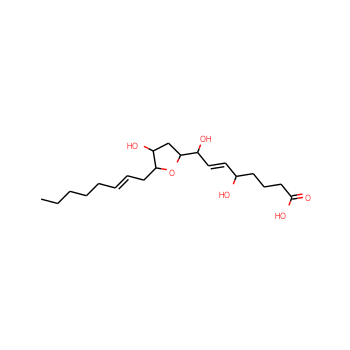 | Level3=InSilico-Generic | *isofurans-eicosanoids/Fatty acids* |
| 291_pos | 663.45282 | 11.51 | C_20_H_32_O_3_^@^ | 13-HETE | 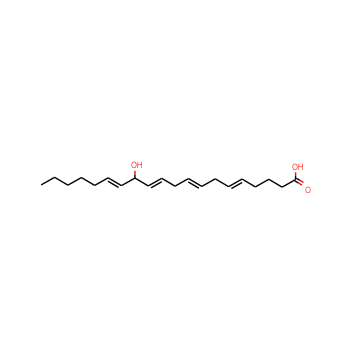 | Level3=InSilico-Generic | *Hydroxy-hydroperoxyeicosatetraenoic acids / Fatty acids* |
| 298_pos | 663.45337 | 13.55 | C_20_H_32_O_3_^@^ | 16(R)-HETE | 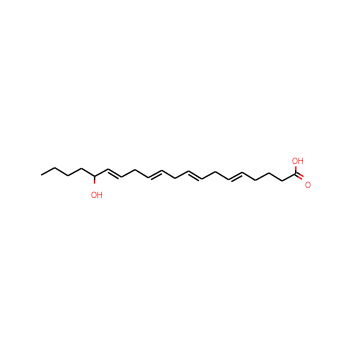 | Level3=InSilico-Generic | *Hydroxy-hydroperoxyeicosatetraenoic acids / Fatty acids* |
|  |  |  |  | **Class IVPP** |  |  |  |
|  |  |  |  | *C18* |  |  |  |
| 823_neg | 583.21844 | 7.59 | C_31_H_36_O_11_ | [13,18-Diacetyloxy-5-hydroxy-10-(2-hydroxypropan-2-yl)-7-methyl-4-oxo-3,15-dioxapentacyclo[10.6.0.01,5.06,10.013,16]octadec-6-en-11-yl] benzoate | 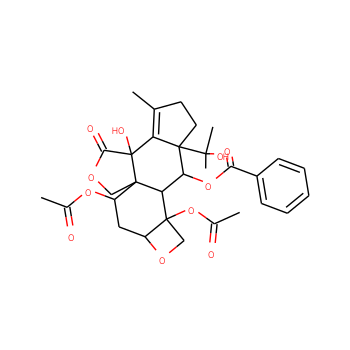 | Level3=InSilico-Generic | *Abeotaxane diterpenoids / Terpenoids* |
| 783_neg | 555.22327 | 6.97 | C_30_H_36_O_10_ | Hainangranatumin C | 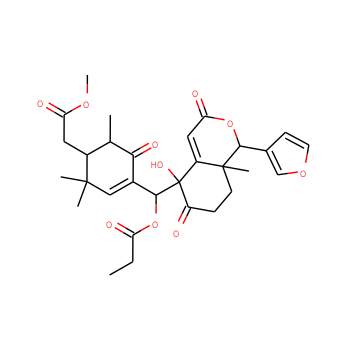 | Level3=InSilico-Generic | *Limonoids / Terpenoids* |
| 691_pos | 397.16483 | 6.95 | C_21_H_26_O_6_^§^ | Strobilurin I | 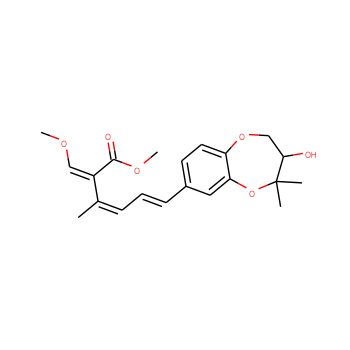 | Level3=InSilico-Generic | *Strobilurins and derivatives / Polyketides* |
| 702_pos | 401.15958 | 7.68 | C_22_H_24_O_7_ | 6-Acetylteuscordin | 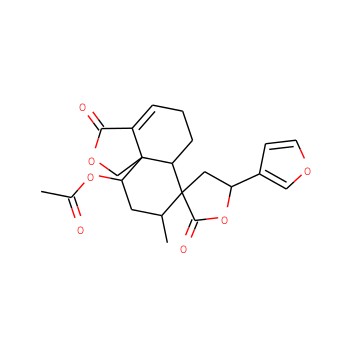 | Level3=InSilico-Generic | *Colensane and Clerodane diterpenoids /Terpenoids* |
| 364_pos | 249.11227 | 6.89 | C_7_H_8_O_2_^‡^ | 2-Methylresorcinol | 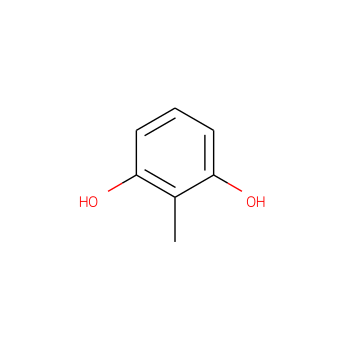 | Level3=InSilico-Generic | *Resorcinol/Shikimates and Phenylpropanoids* |
| 446_neg | 361.16577 | 6.81 | C_20_H_26_O_6_ | Secoisolariciresinol | 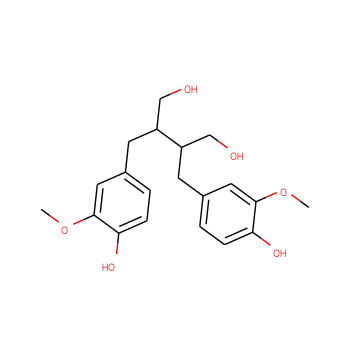 | Level2=MSMSmatch | *Dibenzylbutane lignans / Shikimates and Phenylpropanoids* |
| 225_pos | 194.05943 | 7.11 | C_10_H_10_O_4_^†^ | Ferulate | 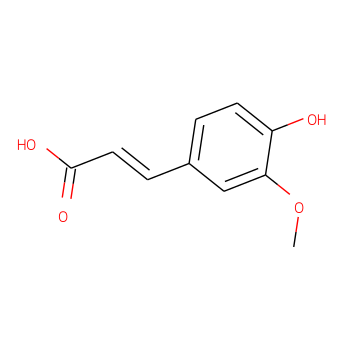 | Level4=MSMSanalog | *Cinnamic acids and derivatives/* *Shikimates and Phenylpropanoids* |
| 224_pos | 193.08583 | 5.73 | C_11_H_14_O_4_^¥^ | 4-hydroxy-3,5-dimethoxy-cinnamyl alcohol (Sinapyl alcohol) | 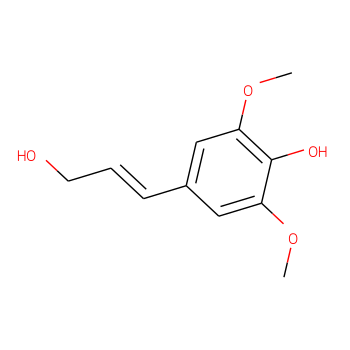 | Level2=MSMSmatch | *Cinnamic acids and derivatives/* *Shikimates and Phenylpropanoids* |
|  |  |  |  | *HILIC* |  |  |  |
| 222_pos | 178.10756 | 4.91 | C_7_H_15_NO_4_ | *2-amino-4,5-dihydroxy-3,4-dimethylpentanoic acid* | 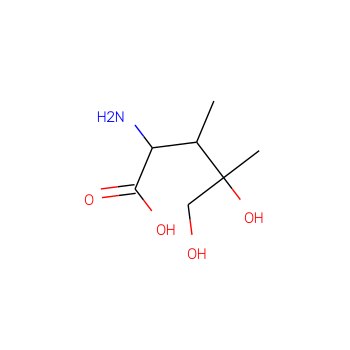 | Level3=InSilico-Generic | *Aminoacids /Amino acids and Peptides* |
|  |  |  |  | *LIPIDOMIC* |  |  |  |
| 411_pos | 764.61896 | 13.43 | C_42_H_83_O_8_P^&^ | *PMeOH 38:0* | 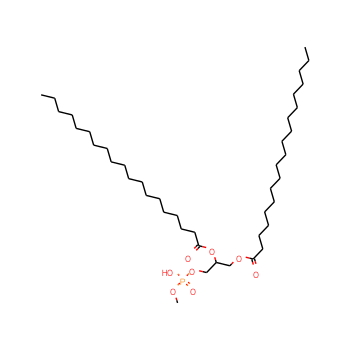 | Level4=MSMSanalog | *Glycerophosphates/fatty acids* |
| 496_pos | 846.75378 | 16.83 | C_53_H_96_O_6_^&^ | *[1-[5-(1-hydroxytridecyl)oxolan-2-yl]-13-(2-methyl-5-oxo-2H-furan-4-yl)tridecyl] octadec-9-enoate* | 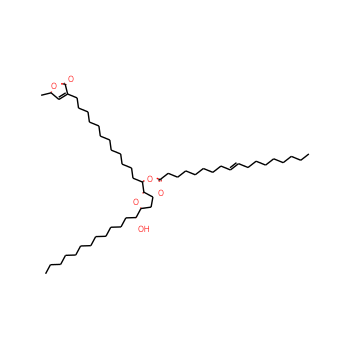 | Level3=InSilico-Generic | *Acetogenins / Polyketides* |
| 36_pos | 188.07071 | 0.82 | C_11_H_11_NO_3_^†^ | *DL-Indole-3-lactic acid* | 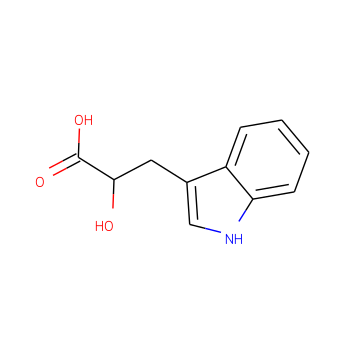 | Level2=MSMSmatch | *Simple indole alkaloids / Alkaloid* |
